# Impact of Blood or Erythrocyte Membrane Fatty Acids for Disease Risk Prediction: Focusing on Cardiovascular Disease and Chronic Kidney Disease

**DOI:** 10.3390/nu10101454

**Published:** 2018-10-07

**Authors:** Oh Yoen Kim, Su Mi Lee, Won Suk An

**Affiliations:** 1 Department of Food Science and Nutrition, Dong-A University, Busan 49315, Korea; oykim@dau.ac.kr; 2 Center for Silver-targeted Biomaterials, Brain Busan 21 Plus Program, Graduate School, Dong-A University, Busan 49315, Korea; 3 Department of Internal Medicine, Dong-A University, Busan 49201, Korea; promise131@hanmail.net

**Keywords:** fatty acid, cardiovascular disease, chronic kidney disease, saturated fatty acid, monounsaturated fatty acid, omega-3 fatty acid, omega-6 fatty acid, *trans*-fatty acid

## Abstract

Fatty acids (FAs) are essential nutrients and main constituents of cell membranes that are involved in the signaling pathway and associated with health conditions. We investigated if blood or erythrocyte membrane FAs can predict the risk of cardiovascular disease (CVD), chronic kidney disease (CKD), and related complications. Omega-3 (*n*-3) FAs are important predictors for metabolic syndrome, diabetes, CVD, and CKD risks, and the *n*-3 index is also a good biomarker for sudden cardiac death in coronary artery disease. Linoleic acid, which is one of the major *n*-6 FAs reflecting recent dietary FA intake, may predict CVD risk and mortality in the general population and patients with CKD. Monounsaturated FAs (MUFAs) are also related to diabetes or diabetic nephropathy. Oleic acid, a major MUFA, is an emerging marker that is related to acute coronary syndrome, low glomerular filtration rate, and vascular calcification in patients with CKD, and can be modified by *n*-3 FA supplementation. Saturated FAs, *trans*-FAs, and FA desaturation/elongation are associated with CVD risk; however, few studies have been conducted on patients with CKD. In summary, blood or erythrocyte membrane FA measurements are important for CVD and CKD risk prediction and management. Further studies are needed to elucidate the FAs for their risk predictions.

## 1. Introduction

Fatty acids (FAs) are one of the important energy sources and membrane constituents and they play essential roles in metabolic homeostasis through their functional properties being involved in the signaling pathways of the body [1,2]. The cell membrane, including the mitochondria, is composed of several FAs forming the lipid bilayer; thus, changes in FA contents may affect fluidity and affinity of receptors on the cell membrane and transport of ion and consequently cause cell senescence, apoptosis, or autophagy [3,4]. Therefore, changes in FA contents of the cell membrane or circulating blood may be closely involved in several disease conditions. The prevalence of chronic kidney disease (CKD) is increasing, owing to the increased proportion of elderly individuals and patients diagnosed with obesity, hypertension, metabolic syndrome (MetS), diabetes, and cardiovascular disease (CVD). CVD is not only a result of CKD, but also an important cause of CKD. However, the impact of FA measurement in the blood or erythrocyte membrane for the prediction of CVD and CKD risks is not clearly elucidated.

The FA compositions in blood cholesteryl esters, phospholipids, or erythrocytes reflect the dietary FA composition during the recent 1–3 months, as well as the endogenous conversion of ingested FAs by desaturation and/or elongation [5,6]. Both dyslipidemia and hyperglycemia may also be related to the FA compositions of the blood or erythrocyte membrane. It is well known that a higher omega-3 (*n*-3) FA intake was associated with a reduced CVD mortality [7]. For several decades, clinical trials and population-based epidemiological studies have attempted to decipher the effect of dietary fats and blood or tissue FA, and their combination effect on metabolic disorders, such as MetS and diabetes, and CVD and its complications, including CKD [8,9,10,11]. However, the results are still controversial. In addition, there are few studies investigating the entire contents of FAs, saturated FA (SFA), monounsaturated FA (MUFA), *n*-3 FA, *n*-6 FA, and *trans*-FA (TFA) [12]. Therefore, in this review, we aimed to investigate whether blood or tissue FAs can be a useful predictor for the risk of CVD, CKD, and related complications.

## 2. Impact of Dietary FAs on CVD Risk

Dietary guidelines emphasize both the quantity and quality of dietary fat for good health; for example, the National Cholesterol Education Program-Third Adult Panel (NCEP-ATPIII) guideline [13], American College of Cardiology (ACC)/American Heart Association (AHA) Cholesterol Guideline [14], Dietary Reference Intake for Koreans (KDRIs) [15], and guideline for the management of dyslipidemia by the Korean Society of Lipid and Atherosclerosis in 2015 [16] recommend individuals to consume SFAs < 7% of the total calorie intake (TCI). These dietary guidelines also suggest the consumption of at least 1 or 2 oily fishes per week, which provides 250–500 mg of docosahexaenoic acid (DHA, C22:6*n*-3) and eicosapentaenoic acid (EPA, 20:5*n*-3) per day. In addition, the KDRIs recommend a total dietary fat consumption between 15% and 30% of the TCI [15].

Dietary fat intake is related to circulating lipid profiles and CVD-related risk. According to previous reports, increased SFA intake in relation to TCI is associated with increased total cholesterol and low-density lipoprotein (LDL) cholesterol levels in the blood by reducing LDL receptor activities in the cells and tissues [17]; it also increases inflammatory responses by increasing lipopolysaccharide uptake in the intestine to access the blood stream easily [18,19]. This finding was also partly supported by the report by Na et al. [20], showing that individuals that were consuming SFAs ≥ 7% of the TCI showed significantly higher levels of neutrophil gelatinase-associated lipocalin (NGAL), one of the inflammatory markers and early urinary biomarkers of tubulointerstitial injury, than those consuming SFAs < 7% of the TCI.

A number of longitudinal and prospective cohort studies have also reported that *n*-3 FA intake can be beneficial for CVD mortality reduction [21,22,23]; blood pressures (BPs); and, circulating triglyceride levels were significantly reduced by daily consumption of ≥3 g of *n*-3 long-chain polyunsaturated FAs (LC-PUFAs) in the form of fish oil, particularly in middle-aged and elderly individuals [23,24]. Conversely, diastolic BP was lowered by daily intake of DHA increasing up to 0.7 g in the short term; however, the endothelial function was not altered [25]. A cross-sectional study showed no significant relationship between *n*-3 PUFA intake and brachial artery flow-mediated dilation (ba-FMD) [26]. In addition, the endothelial function expressed by BPs, ba-FMD, and arterial stiffness were not improved by daily *n*-3 PUFA (1.3–3.0 g) injection for 1–3 months in young and middle-aged individuals [27,28] or by daily consumption of ≤1 g of *n*-3 PUFAs for a year in healthy adults [21].

A meta-analysis and systematic review showed that a six-month consumption of a high MUFA diet (>12% of the total energy content) effectively and significantly reduced the fasting glucose levels and glycated hemoglobin percentages in adults with cardiometabolic risks, such as type 2 diabetes, impaired glucose tolerance, insulin resistance (IR), overweight, or obesity [29,30]. However, the effect of MUFA consumption on cardiometabolic risks is still controversial among studies; therefore, further evidences from long-term clinical studies and large-scale population-based studies are needed.

A high dietary intake of TFA was associated with a higher risk of CVD in large cohort studies [12,31]. TFAs induce dyslipidemia, including increased LDL cholesterol and decreased high-density lipoprotein (HDL) cholesterol levels [32]. Therefore, the Food and Drug Administration in the United States obliges food manufacturers and fast-food restaurants to indicate the TFA content in food labels, and the KDRIs also recommend the consumption of TFAs < 1% of the TCI per day [15].

Based on the findings of previous reports, we summarized that the quality and quantity of dietary fat intake reflect the circulating lipid profiles and CVD risk-related markers (Table 1), and cautiously suggest that consumption of SFAs < 7% and TFAs < 1% of the TCI, respectively, within the recommended range of the total fat intake in relation to the TCI might be beneficial for cardiovascular health. In addition, regarding supplementary PUFA intake, the consumption of LC-PUFAs ≥ 3 g for ≥3 months would be effective in the reduction of CVD risk, including lowering of the circulating triglyceride levels in healthy individuals; however, further evidence from large-scale long-term clinical trials is needed to elucidate the optimal amount of consumption for cardiovascular health.

## 3. Impact of Dietary FAs on CKD Risk Prediction

Albuminuria with a normal glomerular filtration rate (GFR) or a GFR of <60 mL/min/1.73 m^2^ without albuminuria indicates CKD, and both albuminuria and a low GFR also explain an increased risk of CVD [39]. In fact, increased proteinuria, uncontrolled hypertension, and sustained high glucose levels in diabetes and dyslipidemia are well-known risk factors for CKD progression [40,41,42,43]. Many studies have reported the importance of dietary fat in CKD and CKD progression and have shown the association between dietary fat content and kidney function assessed by albuminuria or the GFR [33,34,35]. A lower intake of PUFAs, linoleic acid (LA, C18:2*n*-6) and α-linolenic acid (ALA, 18:3*n*-3), is related to CKD (24-h urinary albumin excretion of >30 mg and/or GFR of <60 mL/min/1.73 m^2^) in patients with type 2 diabetes [33,34]. Significantly higher intake of PUFAs and lower intake of MUFAs were found in patients with diabetes and CKD than in patients without CKD in a cross-sectional study [34]. On the contrary, ALA intake was associated with CKD in the Blue Mountains Eye Study with a total of 2600 participants aged ≥50 years [35]. Increased dietary intake of *n*-3 FA and fish significantly reduced the odds ratio of having CKD; however, the FA contents were not measured in this study [35].

A Mediterranean diet was associated with a decreased incidence of a GFR of <60 mL/min/1.73 m^2^ in the Northern Manhattan Study that enrolled 900 participants with a nearly normal baseline GFR [11]. However, the risk of hyperkalemia should be notified in patients with advanced CKD or those undergoing dialysis. In patients with diabetes, some studies have reported a positive relationship between the progression of albuminuria and dietary SFA consumption [33,38]. A higher animal fat intake was positively associated with the presence of albuminuria, while higher intakes of SFA, MUFA, and animal fat were associated with a decreased estimated GFR in 3448 women during the 11 years of follow-up of the Nurses’ Health Study [36]. In particular, animal fat was highly correlated with SFA in this study. An inverse relationship between low-fat dairy food consumption and microalbuminuria was reported in the Multi-Ethnic Study of Atherosclerosis (MESA) [44]. A higher SFA intake was also significantly associated with a high incidence of albuminuria in 19,256 participants of the Reasons for Geographic and Racial Differences in Stroke study [37]. In a subgroup analysis, the consumption of SFAs and TFAs was associated with a reduced GFR after adjustment for age and energy intake [37].

Further studies are necessary to elucidate the relationship between dietary FA contents and albuminuria or CKD incidence. It is presumed that consumption of less SFAs and TFAs may prevent or delay the metabolic disturbances that progress GFR decline or microalbuminuria.

## 4. Blood or Tissue FAs as Predictors for the Risks of CVD and CKD

The FA compositions in circulating cholesteryl esters, phospholipids, or erythrocytes reflect the dietary FA composition during the recent 6–12 weeks; particularly, LA and ALA in serum phospholipids are known as biomarkers of long-term essential FA intake [1,2]. Ingested FAs are also endogenously converted to other types of FAs by desaturation and/or elongation [5,6]. Numerous studies, including clinical trials and population-based epidemiological studies, have reported the association of the consumption of dietary fats and blood or tissue FAs with CVD, CKD, and related risks. Herein, we summarized the association between FAs in the blood or tissues and the risk of CVD and CKD, focusing on the role of FAs in the diagnosis and prognosis of CVD and CKD.

### 4.1. Impact of PUFAs on CVD Risk

*n*-3 PUFAs were reported to reduce the risk for CVD by modulating the established risk factors (i.e., dyslipidemia, high BP, central obesity, and inflammation) through multiple relevant molecular pathways [45]. The risk of type 2 diabetes was negatively associated with erythrocyte membrane *n*-3 PUFA in a cross-sectional study comparing age- and sex-matched controls [46]. According to previous reports [45,47,48], the proportions of *n*-3 PUFAs, particularly DHA in serum phospholipids, were significantly lower in patients with coronary artery disease (CAD) and particularly in those with MetS than in controls without CAD [22]. The proportion of DHA in circulating phospholipids was also inversely associated with CVD risk parameters and arterial stiffness expressed by the brachial-ankle pulse wave velocity (baPWV) in metabolically healthy men [48]; conversely, a higher proportion of DHA in the erythrocytes was associated with improved endothelial function, especially in young men who had some features of IR [6]. In a previous report, DHA and EPA were found to be important FAs for distinguishing between intracranial atherosclerotic stenosis (ICAS) and no cerebral atherosclerotic stenosis among patients with stroke [49]. Particularly, the risk of ICAS was inversely associated with the levels of DHA in blood phospholipids, indicating that the risk might be increased at lower levels of DHA. It may indicate that sufficient amounts of DHA in the plasma may reduce the risk of ICAS.

The *n*-3 index, defined as the sum of EPA and DHA contents in the erythrocyte membrane, is a potential risk factor for sudden cardiac death from CAD [50]. This index was closely related to EPA and DHA contents in the cardiac tissue and blood phospholipid [50,51,52]. An *n*-3 index of >8% was associated with protection from CAD mortality as compared with an index of <4% in a 10-cohort meta-analysis [53]. A recent report showed that PUFAs in the red blood cells reflect the phospholipid PUFA composition of major organs in mice [54]. Therefore, the erythrocyte membrane FA contents can be an important biomarker for CVD risk.

The level of LA, an essential *n*-6 FA in serum phospholipids, was significantly lower in patients with CAD patients and particularly in those with MetS than in controls without CAD [22]. Conversely, the levels of arachidonic acid (C20:4*n*-6), dihomo-γ-linolenic acid (DGLA, C20:3*n*-6), and *n*-6/*n*-3 PUFAs were higher in the serum phospholipids of patients with CAD than in those of healthy controls [22]. Further, LA was negatively associated and DGLA was positively associated with arterial stiffness in healthy controls [6]. Interestingly, Iggman et al. [55] showed, for the first time, that adipose tissue LA, one of the most predictable biomarkers for dietary *n*-6 PUFA, was associated with a lower all-cause mortality, with a tendency toward a lower CVD mortality through their 15 year-prospective cohort study.

Numerous in vitro and in vivo studies have been performed to elucidate how PUFAs, particularly *n*-3 PUFAs in the circulation, are involved in the mechanisms against atherosclerotic processes; *n*-3 PUFAs incorporate to membrane phospholipids and alter the physicochemical properties of membrane structures, thereby making membrane-associated protein easily localized [45,56] and modulating cellular inflammatory processes and cell growth or apoptosis through the mitogen-activated protein kinase signaling or nuclear factor κB pathway [45,57,58,59,60]. Particularly, DHA and EPA, which are the main *n*-3 PUFAs, significantly reduce the key regulators for cytokine transcription from circulating immune cells, thereby attenuating the production of inflammatory cytokines, such as interleukin (IL)-1β and IL-6 [61]. However, there are still controversial or contrary results [62].

Taken together, PUFAs, particularly *n*-3 FAs in circulating phospholipids or erythrocytes, may be an indicator for CVD risk and play a protective role against atherosclerotic pathogenesis by controlling inflammation and oxidative stress in blood and membrane conditions. However, as most studies have been performed on circulating phospholipids, further studies on erythrocytes and tissues should be conducted to elucidate the threshold of PUFAs, which would reflect the status of CVD risk.

### 4.2. Impact of SFAs, MUFAs, FA Desaturation/Elongation and TFA on CVD Risk

As mentioned above, a higher consumption of SFA is associated with abnormal lipid profiles and pro-inflammatory responses not only in the circulation, but also in the tissues [15,17,18]. Similarly, SFA composition in serum phospholipids was positively associated with triglyceride levels in individuals with MetS; for example, high levels of palmitic acid (PA, 16:0) in circulating phospholipids were observed in IR and MetS patients [47,63].

**Figure 1:**
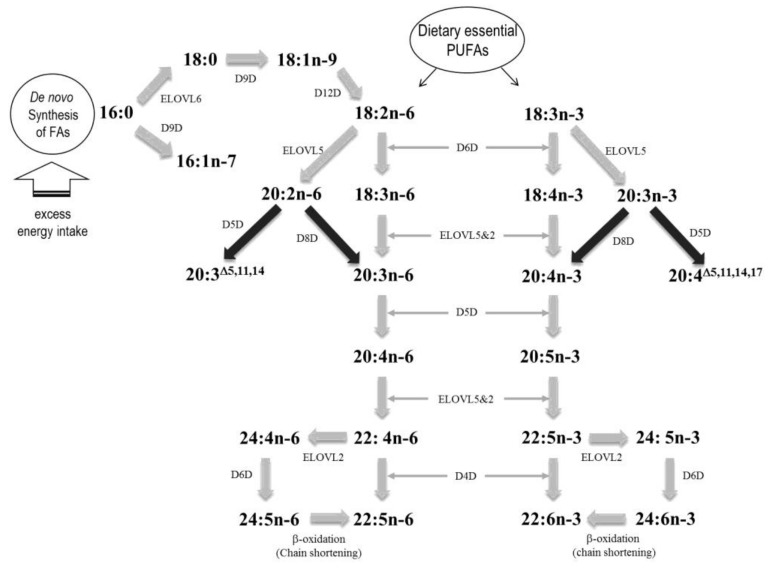
Fatty acids elongation and desaturation pathways. PUFAs, polyunsaturated fatty acids; FAs, fatty acids.

MUFA was also associated with the onset of type 2 diabetes and CVD risk [64,65,66,67,68]. According to the report by Cho et al. [63], total MUFA, oleic acid (OA, 18:1*n*-9), palmitoleic acid (16:1*n*-7), and Δ-9-desaturase (D9D, 18:1*n*-9/18:0 or 16:1*n*-7/16:0) activity were significantly associated with early alteration of the fasting glycemic status and suggested as useful markers for predicting the risk of type 2 diabetes and cardiometabolic diseases. The risk of type 2 diabetes was associated with erythrocyte membrane PA, OA contents, and Δ-6 desaturase (D6D, 18:3*n*6/18:2*n*6) and D9D (18:1*n*9/18:0) activities [46]. D9D is known as a rate-limiting enzyme that is responsible for converting SFA to MUFA, e.g., PA and stearic acid (C18:0) to palmitoleic acid and OA, respectively [63,69] (Figure 1). In this aspect, D9D could be thought as a cell protector against lipotoxicity caused by over-accumulated SFAs; however, the products of D9D can also be the substrates for lipid synthesis (i.e., triglycerides, cholesterol esters, and phospholipids), as well as the major lipid components of cell membranes [24,48,65]. According to the report by Ortinau et al. [69], inhibition of stearoyl-CoA desaturase 1 (SCD1, a mouse isoform of D9D) in obese mice improves glucose and insulin tolerance and attenuates hepatic inflammation; however, these were not observed in lean mice. A prospective cohort study also showed interesting results that palmitoleic acid in the adipose tissue is significantly associated with an increased mortality, whereas heptadecanoic acid (17:0) in the adipose tissue was associated with decreased mortality [12,47]. Therefore, higher conversion from SFAs to MUFAs by D9Ds might contribute to the development of type 2 diabetes and CVD, which are strongly linked to obesity and IR [45,61].

In addition, the activities of desaturating enzymes, such as Δ-5-desaturase (20:4*n*-6/20:3*n*-6), are decreased, while those of D6D and D9D are increased in patients with obesity and MetS as compared with healthy controls [45,61]. The activity of DGLA/LA, including that of elongase 5, which indicates elongation from 18:3*n*-6 to 20:3*n*-6, as well as that of D6D, also increased in individuals with metabolically unhealthy conditions [59]. Higher plasma phospholipid levels of the *trans*-isomers of LA (*trans*-18:2) were associated with a higher risk of fatal CAD and sudden cardiac death in the elderly [70]. In contrast, higher levels of the *trans*-isomers of OA (*trans*-18:1) were associated with a lower risk of sudden cardiac death in this study. Higher erythrocyte membrane *trans*-18:2 contents were also associated with sudden cardiac death [71]. Further studies are needed to investigate the effects of *trans*-18:1 and *trans*-18:2 for CVD risk prediction.

Increased D9D activity and highly accumulated MUFAs, as well as higher proportions of SFAs and *trans*-18:2 in the circulation and erythrocyte membrane, may be important indicators for CVD risk. Particularly, desaturase or elongase activities estimated by the FA ratio can be a convenient indicator for CVD-related risk.

### 4.3. Impact of PUFAs on CKD Risk Prediction and Renal Progression

Although the association between dietary fat intake and kidney function has been reported, investigations with measurements of FA contents in human biological specimens are limited [72,73,74]. In an Italian population-based cohort study with a three-year follow-up, participants with a higher GFR had higher levels of total PUFAs, *n*-3 FAs, and *n*-6 FAs in the plasma among 931 elderly subjects [72]. The plasma PUFA levels at baseline were inversely associated with urine protein excretion in this study. In addition, participants with lower plasma PUFA levels at baseline had a higher risk of developing renal insufficiency (creatinine clearance rate of <60 mL/min) among 398 participants with a creatinine clearance rate of >60 mL/min during the three-year follow-up. Notably, only the *n*-3 FA levels were inversely associated with the risk of developing renal insufficiency or death in this study. In a randomized, placebo-controlled, two-period crossover trial that employed 4 g/day of *n*-3 FA supplementation, increased contents of EPA and DHA in the erythrocyte membrane and decreased *n*-6-to-*n*-3 FA ratio were found in patients with diabetic nephropathy [73]. There were significant decreases in the 24-h urinary excretion of albumin, NGAL, and liver FA-binding protein (LFABP) among participants taking renin-angiotensin-aldosterone system blockers. NGAL and LFABP are early biomarkers of tubulointerstitial injury [75,76]. This study supports that higher contents of *n*-3 FAs in the erythrocyte membrane reflect less tubulointerstitial injury. We also speculate that lower contents of *n*-3 FAs in the erythrocyte membrane may be related to diabetic nephropathy. After kidney transplantation, patients with low levels of *n*-3 FAs in the blood showed faster graft dysfunction than those with high levels of *n*-3 FAs [74]. Lower levels of *n*-3 PUFAs in the plasma were positively associated with the development of interstitial fibrosis during the first year after transplantation [77]. Therefore, circulating *n*-3 FA levels may be a good indicator for kidney function after kidney transplantation. In a recent study, *n*-3 FA supplementation attenuated the progression of albuminuria in subjects with type 2 diabetes and CAD [78]. Although this study did not measure the FA contents in the blood or tissues, it can be assumed that individuals without the attenuated progression of albuminuria may have increased EPA and DHA contents. Therefore, the measurement of FA contents may reinforce the study quality, especially in intervention studies.

### 4.4. Impact of MUFAs on CKD Risk Prediction

The FA contents in the erythrocyte membrane at baseline were compared between healthy volunteers and patients with diabetes in an *n*-3 FA supplementation study [79]. Patients with diabetes showed higher OA contents in the erythrocyte membrane than healthy controls. We speculate that higher OA contents in the erythrocyte membrane might be related to diabetes or diabetic nephropathy. The plasma OA level increased with reduced kidney function in 29 patients with CKD stage 3–5 as compared with that in 10 control subjects [80]. Monounsaturated cis-vaccenic acid (18:1*n*-7) in plasma phospholipids was associated with a GFR of <60 mL/min/1.73 m^2^ in the 2792 participants from the MESA [81]. However, there are few data explaining that SFAs, FA desaturation/elongation, and TFA contents are related to albuminuria and CKD incidence. Further studies are needed to elucidate the relationship between blood or erythrocyte FAs and albuminuria or CKD incidence.

### 4.5. Impact of FAs on CVD Risk Prediction and Mortality in Patients with CKD

CVD is commonly observed in patients with CKD and the mortality rate is high within the first 90 days of dialysis [82]. In patients undergoing dialysis, DHA in the erythrocyte membrane is inversely associated with disease mortality [83,84]. The OA content in the erythrocyte membrane was increased in patients with acute coronary syndrome or in those undergoing dialysis [85,86,87,88]; moreover, *n*-3 FAs can reduce OA levels in patients undergoing dialysis [89,90]. It is presumed that erythrocyte MUFAs, including OAs, might be related to CVD risk, and *n*-3 FAs can favorably control the FA contents in the erythrocyte membrane under uremic conditions. The blood levels of SFA and MUFA are higher, and the levels of PUFA are lower in patients undergoing hemodialysis (HD) than in healthy controls; these findings are associated with lipid disorders and cardiomyopathy [91]. In patients with sudden cardiac death during the first year of HD, sudden cardiac death has a positive relationship with the serum levels of SFA and a negative relationship with those of *n*-3 FA; especially, it showed an inverse relationship with the levels of DHA [92,93]. Among patients undergoing HD with established CVD, the serum phospholipid DHA levels were significantly lower in patients with atrial fibrillation than in those with sinus rhythm [94]. Arteriosclerosis in HD is also an important risk factor for CVD. The baPWV and blood DHA levels had a negative association in patients without diabetes undergoing HD [95]. In Swedish patients undergoing dialysis, the plasma LA levels were inversely related to systemic inflammatory markers, such as IL-6, and all-cause mortality [96]. Conversely, the *n*-3 FA levels were not associated with mortality in this study. This finding is presumed because of the high intake of *n*-3 FA in the general Swedish population. This study raises the importance of increased consumption of LA-rich food, such as vegetable oils [96].

Free FAs, also known as non-esterified FAs, are essential as energy substrates for the myocardium and can be harmful for cardiac function or structure [97]. Free FA accumulation is associated with CVD risk and mortality. In a prospective cohort study including 1221 elderly men in Sweden, increased free FA levels are associated with the risk for cardiovascular mortality in patients with CKD [98]. Even in patients who underwent kidney transplantation, CVD is still a major cause of post-transplantation mortality. In a Norwegian cross-sectional study that was conducted on 1990 subjects, the plasma *n*-3 FA levels were associated with lower resting heart rate, lower triglyceride level, and higher HDL level, which may be estimated to lower CVD risk [99]. In recipients of renal transplants, the plasma phospholipid *n*-3 FA levels were associated with lower overall and cardiovascular mortalities. In particular, sudden cardiac death and death from stroke were inversely associated with the *n*-3 FA levels in transplanted patients [74].

It is assumed that FA modification via diet or FA supplementation can control CVD risk, including BP, which is a well-known traditional risk factor for cardiovascular mortality in patients undergoing dialysis. It is relatively well known that *n*-3 FAs have a favorable effect on cardiovascular morbidity and mortality, although some studies did not show a reduced CVD risk after *n*-3 FA supplementation [100,101]. In particular, the AHA recommends that patients with CKD and heart diseases consume at least 1 g of *n*-3 FA. Further large-scale prospective studies are necessary.

### 4.6. Impact of FAs on Vascular Calcification Prediction in Patients with CKD

Vascular calcification (VC) reflects vascular aging in the elderly [102]. It also increases the risk of morbidity and mortality and it is commonly found, especially in patients with CKD [103]. This is an active process in which the vascular smooth muscle cells differentiate into osteoblast-like cells; it is induced by lipotoxicity, inorganic phosphate, inflammatory cytokines, and oxidative stress [104,105,106,107]. The reduction of klotho, an aging-related protein, is considered to be one of the causes of VC in CKD [108]. SCD1 is associated with FA synthesis. The lack of klotho inhibits the expression of SCD and eventually leads to VC [109]. A positive relationship between SFA accumulation and VC was reported [110]. Arterial medial calcification induced by warfarin in Sprague-Dawley rats was reduced after EPA supplementation (1 g/kg/day) [111]. Among patients undergoing HD, the OA and MUFA contents in the erythrocyte membrane were significantly higher in patients with significant VC scores than in those without significant VC scores [88]. However, the EPA and DHA contents in the erythrocyte membrane were not different between the two groups. To date, no study has investigated the role of the FA contents in VC in patients that were treated with peritoneal dialysis (PD). However, even in patients undergoing PD, VC occurs frequently, as in those undergoing HD; it can be presumed that there is a change in the FA levels with the presence of VC based on the fact that the contents of erythrocyte membrane FAs, such as SFA and OA, have changed before and after taking *n*-3 FA [90]. Further large-scale prospective studies are needed to evaluate the role of the FA contents in VC in patients with CKD.

## 5. Conclusions

Dietary intake is surveyed as one of the major health assessment parameters; however, it may not always accurately reflect the status of chronically ill patients or elderly individuals. Many previous studies have reported the association of the FA contents in the blood or erythrocyte membrane with dietary FA intake or the risk of CVD and CKD, as summarized in Table 2. The properties of FAs (i.e., chain length, desaturation, or saturation) in blood or erythrocyte membrane affect cell membrane fluidity, affinity of the receptors, transport of ions, oxidative stress, and inflammatory response, which are closely associated with cell death and survivals. Consequently, these phenomena can directly have effects on vascular condition such as blood flow, glycemic status, stiffness, and calcification. Notably, measurements of FAs in the blood or erythrocyte membrane provide information on the essential FAs that are needed in deficient conditions as well as on avoidance of high contents of unfavorable FAs.

**Figure 2:**
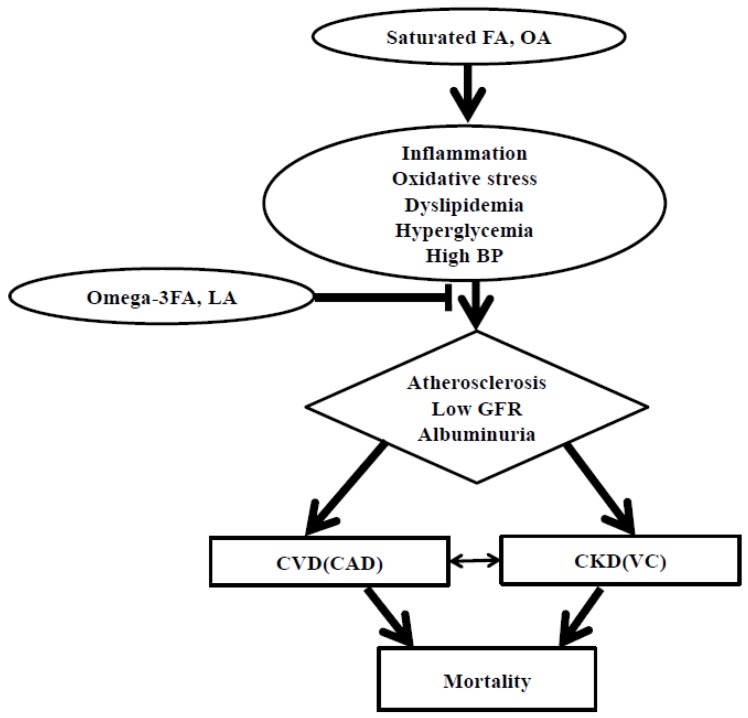
Possible mechanisms: blood or erythrocytes fatty acids as an indicator for cardiovascular disease and chronic kidney disease risk prediction. FA, fatty acids; OA, oleic acid; BP, blood pressure; LA, linoleic acid; GFR, glomerular filtration rate; CVD, cardiovascular disease; CAD, coronary artery disease; CKD, chronic kidney disease; VC, vascular calcification.

Therefore, FA measurements in the blood or erythrocyte membrane can be useful for prediction and management of CVD and CKD risks. This review presents that *n*-3 FAs and LA in the blood or erythrocyte membrane can be used as positive parameters that play beneficial roles in the reduction of CVD and CKD risks, but SFA and OA contents can be negative parameters which increase the risks of CVD and CKD (Figure 2). This review will be the base for future studies confirming the FAs for the prediction of the risk of CVD, CKD, and related complications.

## Figures and Tables

**Table 1:** Impact of dietary fatty acids (FAs) on cardiovascular disease (CVD) risk and chronic kidney disease (CKD).

	CVD Risk ^φ^	CKD	
		Non-DM	DM
PUFA	BP ↓, TG ↓ [23,24]	-	Albuminuria ↓ [33,34] GFR decline ↓ [33,34]
*n*-6 PUFA ^φφ^	-	-	Albuminuria ↓ [33,34] GFR decline ↓ [33,34]
*n*-3 PUFA	CVD mortality ↓ [7,21,22,23]	-	-
ALA	-	GFR decline ↑ [35]	Albuminuria ↓ [33,34] GFR decline ↓ [33,34]
DHA	diastolic BP ↓ [25]	-	-
MUFA	FBG ↓, HbA1c ↓ [29,30]	GFR decline ↑ [36]	Albuminuria ↓ [33,34] GFR decline ↓ [33,34]
SFA	TC ↑, LDL ↑, LDLR ↓ [17] NGAL ↑ [20]	Albuminuria ↑ [37] GFR decline ↑ [36,37]	Albuminuria ↑ [33,38]
TFA	CVD ↑ [12,31] LDL ↑, HDL ↓ [32]	GFR decline ↑ [37]	-

^φ^ CVD risk includes dyslipidemia, inflammation, diabetes; ^φφ^ *n*-6 PUFA indicates linoleic acids. ALA, α-linolenic acid; BP, blood pressure; CKD, chronic kidney disease; CVD, cardiovascular disease; DHA, docosahexaenoic acid; DM, diabetes mellitus; FBG, fasting blood glucose; GFR, glomerular filtration rate; HDL, high-density lipoprotein cholesterol; HbA1c, glycated hemoglobin; LDL, low-density lipoprotein cholesterol; MUFA, monounsaturated fatty acids; NGAL, neutrophil gelatinase-associated lipocalin; PUFA, poly unsaturated fatty acid; SFA, saturated fatty acids; TC, total cholesterol; TG, triglyceride; TFA, *trans*-fatty acids. Arrows pointing up and down indicate the increase and decreased risks of CVD and CKD, respectively, during study.

**Table 2:** Blood or erythrocyte FAs as predictors for risks of CVD and CKD.

	CVD				CKD			
	DM or DL or MetS	AS	ICAS or CAD	Mortality	Proteinuria or GFR Decline	VC	CVD	Mortality
PUFA					↓ [34,72,73]		↓ [91]	
*n*-6								
LA		↓ [6,48] ^φ^	↓ [49]	↓ [55]	↓ [34]			↓ [96]
*trans*-LA			↑ [70]	↑ [70,71]				
DGLA		↑ [6,48]						
*n*-3	↓ [46]				↓ [74]			↓ [92,93]
ALA					↓ [34]			
DHA	↓ [46]	↓ [48] ^φ^	↓ [49]			↓ [95] ^φ^	↓ [94]	↓ [83,84,92,93]
EPA	↓ [46]					↓ [111]		
*n*-3 index	↓ [46]			↓ [50,53]				
MUFA	↑ [46,63]					↑ [88]	↑ [91]	
OA	↑ [46,63]		↑ [53]			↑ [88]		
*trans*-OA				↓ [70]				
Palmitoleic acid	↑ [46,63]							
SFA	↑ [18,63]					↑ [110]	↑ [91]	↑ [92,93]
Δ-6-desaturase	↑ [46,63]							
Δ-9-desaturase	↑ [46,63]							

^φ^ Pulse wave velocity was used to evaluate the AS or VC. ALA, α-linolenic acid; AS, Arterial stiffness; CAD, coronary artery disease; CKD, chronic kidney disease; CVD, cardiovascular disease; DM, diabetes mellitus; DGLA, dihomo-γ-linolenic acid; DHA, docosahexaenoic acid; DL, dyslipidemia; EPA, eicosapentaenoic acid; GFR, glomerular filtration rate; ICAS, intracranial atherosclerotic stenosis; LA, linoleic acid; MUFA, monounsaturated fatty acids; MetS, metabolic syndrome; *n*-3 index, omega-3 index; OA, oleic acid; PUFA, poly unsaturated fatty acid; SFA, saturated fatty acids; VC, vascular calcification. Arrows pointing up and down indicate the increase and decreased risks of CVD and CKD, respectively, during study.
